# Influenza vaccination during pregnancy: a qualitative study of the knowledge, attitudes, beliefs, and practices of general practitioners in Central and South-Western Sydney

**DOI:** 10.1186/1471-2296-15-102

**Published:** 2014-05-23

**Authors:** Louise Maher, Angela Dawson, Kerrie Wiley, Kirsty Hope, Siranda Torvaldsen, Glenda Lawrence, Stephen Conaty

**Affiliations:** 1 NSW Public Health Officer Training Program, NSW Ministry of Health, 73 Miller St, North Sydney, NSW 2060, Australia; 2 School of Public Health and Community Medicine, University of New South Wales, High St, Kensington, NSW 2052, Australia; 3 Health Services and Practice Research Group, Faculty of Health, University of Technology, 15 Broadway, Ultimo, NSW 2007, Australia; 4 National Centre for Immunisation Research and Surveillance, Locked bag 4001, Westmead, NSW 2145, Australia; 5 Discipline of Paediatrics & Child Health, The University of Sydney, NSW 2006, Australia; 6 Sydney and South Western Sydney Local Health Districts Public Health Unit, PO Box 374, Camperdown, Sydney 1450, Austraila

**Keywords:** Influenza, Vaccine, Pregnancy, General practitioners, Recommendation

## Abstract

**Background:**

Pregnant women have an increased risk of influenza complications. Influenza vaccination during pregnancy is safe and effective, however coverage in Australia is less than 40%. Pregnant women who receive a recommendation for influenza vaccination from a health care provider are more likely to receive it, however the perspectives of Australian general practitioners has not previously been reported. The aim of the study was to investigate the knowledge, attitudes, beliefs, and practices of general practitioners practicing in South-Western Sydney, Australia towards influenza vaccination during pregnancy.

**Methods:**

A qualitative descriptive study was conducted, with semi-structured interviews completed with seventeen general practitioners in October 2012. A thematic analysis was undertaken by four researchers, and transcripts were analysed using N-Vivo software according to agreed codes.

**Results:**

One-third of the general practitioners interviewed did not consider influenza during pregnancy to be a serious risk for the mother or the baby. The majority of the general practitioners were aware of the government recommendations for influenza vaccination during pregnancy, but few general practitioners were confident of their knowledge about the vaccine and most felt they needed more information. More than half the general practitioners had significant concerns about the safety of influenza vaccination during pregnancy. Their practices in the provision of the vaccine were related to their perception of risk of influenza during pregnancy and their confidence about the safety of the vaccine. While two-thirds reported that they are recommending influenza vaccination to their pregnant patients, many were adopting principles of patient-informed choice in their approach and encouraged women to decide for themselves whether they would receive the vaccine.

**Conclusions:**

General practitioners have varied knowledge, attitudes, and beliefs about influenza vaccination during pregnancy, which influence their practices. Addressing these could have a significant impact on improving vaccine uptake during pregnancy.

## Background

Pre-natal influenza infection is associated with an increased risk for both mother and baby, including respiratory and cardio-pulmonary hospitalisation, pre-term delivery, fetal distress, and in some cases death [1-7]. These risks are compounded when the mother has comorbidities such as asthma or diabetes, or is infected with a pandemic strain of the virus [4,8]. The most effective strategy for preventing influenza in pregnant women is immunisation, and benefits for both the mother and the infant have been demonstrated, with maternal immunisation significantly reducing respiratory illnesses in both the women and their infants in the first six months of life [9-11]. Influenza vaccination during any trimester of pregnancy is considered safe for both the mother and the foetus [12-15]. It is recommended that all pregnant women in Australia receive the vaccine, [16-18] and these guidelines have been in place for more than six years. The vaccine is available free of charge to pregnant women in Australia. However, the proportion of pregnant women who receive the vaccine is low, with coverage between 10 and 40% [19-23].

While many factors may influence influenza vaccine uptake during pregnancy, it has been identified that pregnant women who receive a recommendation for the vaccine from a health care provider are more likely to receive the vaccine [22,24-27]. However, antenatal care providers do not routinely recommend influenza vaccination to their pregnant patients: three recent surveys of pregnant and post-partum women in Australia found that only 21–41% of women could recall receiving such a recommendation [21-23]. Where a recommendation was given, approximately half were by a general practitioner [22,23].

Antenatal care providers are more likely to recommend the influenza vaccine to pregnant women if they have good knowledge about influenza and influenza vaccination during pregnancy, positive attitudes towards influenza vaccination during pregnancy, have observed serious medical conditions due to influenza, or have personally received the influenza vaccine [22,28-30]. These studies, which investigated the knowledge, attitudes, and practices of antenatal care providers towards influenza vaccination during pregnancy, have mainly relied on data gathered using written survey techniques in antenatal care settings such as a hospital or clinic. No studies have been undertaken in the Australian primary health care context, and little is known about the perceptions of general practitioners who are the main provider group or avenue through whom pregnant women can access influenza vaccination. The attitudes and practices of general practitioners are therefore likely to have a significant influence on influenza vaccine uptake during pregnancy.

In early 2012, strategies to improve awareness about influenza vaccination during pregnancy among general practitioners working in central and south-western Sydney were implemented. The local director of public health with the directors of obstetrics wrote to all general practitioners in the region reminding them of the recommendation to vaccinate pregnant women for influenza, and referring them to a recent evidence-based statement [18]. They were also provided with multiple copies of a brochure designed to encourage women to be vaccinated, [31] and a reminder stamp about the vaccine was placed in patient-held antenatal care cards.

The aim of this study therefore was to investigate the knowledge, attitudes, beliefs, and practices of general practitioners in the Sydney and South-Western Sydney Local Health Districts in Australia towards influenza vaccination during pregnancy, using qualitative methodology. The qualitative approach was used to provide rich information on the perspectives of the general practitioners, to identify their practices regarding influenza vaccination during pregnancy, and their knowledge, attitudes, and beliefs that inform these practices.

## Methods

A qualitative descriptive methodology [32] was used to describe general practitioners knowledge, attitudes, beliefs, and practices regarding influenza vaccination during pregnancy, and to generate knowledge that can be applied to practice.

Purposive sampling [33] was used to select general practitioners to ensure diversity in terms of the location of the general practice, the practice size, and the practitioner’s sex. A matrix was developed to map these characteristics to guide participant selection informed by a detailed list of all 666 general practices in the Sydney and South-Western Sydney Local Health Districts. The matrix fields included size of the practice (a large practice is where there are 3 or more general practitioners), type of local government area (urban and rural) and sex of general practitioners within a practice. From this matrix, 44 general practitioners were selected to be invited to participate in the study, to ensure that sex, practice size and type of local government area were evenly represented. It was estimated that twenty participants would be required to reach saturation, therefore with an estimated participation rate of less than 50%, forty-four general practitioners were selected to be invited. A low participation rate was expected, due to the high workload and competing demands faced by general practitioners which may limit their availability to participate.

The 44 selected general practitioners were initially contacted by letter, and subsequently by telephone, to invite them to participate. A semi-structured interview guide, using open-ended questions, was developed and consisted of the following broad topic sections: reflections on antenatal care in general practice, the risk of influenza during pregnancy, attitudes and practices about influenza vaccination during pregnancy, perceptions of women’s attitudes towards influenza vaccination during pregnancy, and reflections on how to improve and record coverage rates of influenza vaccination during pregnancy. Within each section of the interview topic guide, more detailed questions and specific probes were prepared to allow the discussion to develop. The interview questions were piloted with two non-participating general practitioners and minor modifications were made to question expression. Semi-structured interviews were conducted in person with all consenting general practitioners in their place of practice by the one researcher (LM) in September 2012. The duration of each interview ranged from fifteen to forty-five minutes. Interviews were audio-recorded and transcribed verbatim by an independent transcriber.

Four researchers (LM, KW, AD, KH) conducted the analysis. Transcripts were read and re-read by the four researchers several times who then met to discuss the emergent concepts, themes and issues across the dataset. Consensus was reached on the main themes and categories to be included in the analysis, based on the aim of the study, and a conceptual framework was developed to capture this. The researchers undertook a closer analysis of each transcript using the conceptual framework to direct this. The qualitative research software tool N-Vivo was employed to code the transcripts using a constant comparative method [34] and each researcher generated a diagrammatic model to illustrate their coding and patterns. These models were then synthesised and guided the reporting process.

This project was approved by the Sydney Local Health District Human Research Ethics Committee (RPAH Zone).

## Results

Of the 44 general practitioners selected, 17 agreed to participate in the study: nine females and eight males; ten from small and seven from large practices; and six from Sydney Local Health District and eleven from South Western Sydney Local Health District. The reasons for non-participation were not ascertained.

Thirty main codes were identified, and grouped into three main categories: (1) general practitioners’ risk perception of influenza infection during pregnancy; (2) general practitioners’ knowledge and perceptions about influenza vaccination during pregnancy; and (3) general practitioners’ approach to promoting and providing influenza vaccination during pregnancy.

### (1) General practitioners risk perception of influenza infection during pregnancy

Overall the general practitioners were not concerned about the risks associated with influenza during pregnancy. One-third did not consider influenza during pregnancy to be a serious risk for the mother or the baby. Two-thirds thought that there was an increased risk associated with influenza during pregnancy, and mentioned miscarriage or premature labor as potential consequences. Some thought that the risks of infection were specifically associated with the H1N1 strain of the 2009 pandemic and not other influenza strains. Other general practitioners said that although they were aware that the risks are reported to be higher in pregnant women compared to the general population, they had no direct experience of a pregnant patient contracting influenza and having serious consequences, and that this in turn decreased their perception of the risk. Many did not perceive that pregnancy alone placed a woman in a high-risk category for influenza, and felt that only pregnant women with other co-morbidities, such as respiratory disease or obesity, were at risk of complications.

“I'm aware that if women get the influenza virus during pregnancy complications are much higher, the severity of the influenza is much higher and so we ought to be vaccinating women during pregnancy”.

“I guess the same (risks) as anyone who doesn’t have a pregnancy. Whether it brings on pre-term labour, possibly, but I am not aware of any specific problems directly related to the pregnancy”.

“I think with the number of people (pregnant women) who catch the flu and the number of people who don’t have any problems with it….I see it’s a small amount of risk involved”.

### (2) General practitioners knowledge, attitudes and beliefs about influenza vaccination during pregnancy

The majority of the general practitioners were aware of the recommendations for influenza vaccination during pregnancy, but most were not confident on all aspects of the recommendations, particularly in relation to timing. Some thought the recommendation specified provision during a specific trimester or only during the influenza season. Some wondered why this recommendation had become a priority.

“So I am fully aware that it is recommended that they get their influenza vaccine if they are going to be pregnant in the flu season, particularly second and third trimester”.

“The thing that surprised us is why suddenly there is a push for vaccinating for flu in pregnant woman….most of us are quite surprised that it is recommended”.

“If you look at it throughout the years that we've never given flu needles to pregnant women we haven't run into much significant problems”.

Most general practitioners identified that influenza vaccination during pregnancy would be beneficial in preventing consequences of infection such as miscarriage or premature labour. Very few specifically nominated the benefits of vaccination for the baby, and when questioned had varied opinions about this.

More than half of the general practitioners had significant concerns about the safety of the vaccine during pregnancy. Many of these general practitioners raised the issue of time – providing the influenza vaccine during pregnancy is a relatively recent practice and they felt that there needed to be a longer period of time where this was practiced without adverse outcomes before they could be confident that the vaccine was completely safe for pregnant women. A number of the general practitioners were concerned that if they provided the vaccine to pregnant women and an adverse event subsequently occurred (which may or may not be related to the vaccine), that women may blame the vaccine and hold the practitioner liable. Some were particularly concerned that the influenza vaccine was rated in product information as Australian Category B2 (drugs which have been taken by only a limited number of pregnant women and studies in animals are lacking but available data shows no evidence of harmful effects on the foetus [35]).

“I’m not fully convinced that it’s totally safe to them, no”.

“I think it is more of an unknown and you tend to be more conservative about what you give [pregnant] patients”.

“With the small amount of risk involved [with influenza] I don’t see that the benefits [of the vaccination] outweigh the risks”.

“My understanding is it category B in pregnancy. Which is a little bit of grey area…. If it was Category A I would be much more likely to recommend it.”

“We have to wait and see whether the information is correct. Most times after a few years you find out that the information might not be that accurate.”

“I just think if they had the flu injection, then whether it was a day, a month, or at any stage after getting the vaccine, that if anything went wrong like foetal death or early labour, I know that they would look at pointing the finger at the flu vaccine as the cause. Whether it is or not. So it is safer as a doctor not to do that”.

The general practitioners who were confident that the vaccine is safe were either more informed about the evidence regarding safety of the vaccine in pregnancy, or were more willing to trust that the vaccine is safe and beneficial based on the fact that it is recommended under the national immunisation guidelines. Most of this group recommended the vaccine to pregnant women and had not observed any adverse outcomes which reinforced their belief that it is safe.

“Yes, well, it was recommended from the health department to do so I would assume that the information is accurate and there's no risk in doing it so I'm happy to follow that”.

“I’ve given it during pregnancy for a few patients and I haven’t noticed anything unusual”.

Few general practitioners were confident about their knowledge regarding influenza vaccination during pregnancy. Most felt that they needed more information; however none reported actively searching to obtain information. Many reported challenges in information management and staying aware of recent research and evidence. Many interviewees specifically asked questions of the interviewer to obtain information about the guidelines and evidence.

“I would take it that somewhere in the world they’ve been vaccinating pregnant women for a reasonably length period and I would probably like to look at some figures in relation to that as to the number of adverse effects that occur and the efficacy of doing it”.

### (3) General practitioners approach to promoting and providing influenza vaccination during pregnancy

The general practitioners’ approach to recommending and providing the influenza vaccine during pregnancy was related to their perception of the risks associated with influenza infection during pregnancy, their confidence in the safety of the vaccine during pregnancy, and also more practical issues such as limited consultation time to cover issues like vaccination with their patients.

Of the two-thirds of those who reported that they recommend the vaccine to their pregnant patients, either intermittently or routinely, the majority recommend the vaccine during the autumn/early winter period, or in a specific trimester (either first or third). Some general practitioners only recommended it to pregnant patients who have higher risks associated with infections due to other conditions such as asthma or chronic disease. A number of general practitioners who were not confident in the safety of the vaccine reported recommending it to patients purely because of the guidelines. The general practitioners who said they do not recommend it were from larger practices, were not registered antenatal shared care providers, and included both male and female general practitioners. Some general practitioners reported challenges in prioritising competing demands during a consultation with a pregnant woman, and that influenza vaccination was often not a high priority, or something that they did not always remember to do.

“If the patient comes to me, and they’re in the first trimester, and it’s winter, I would give it to them”.

“I don’t like the concept of giving a flu vaccine during the first trimester, I would rather wait till later on and that’s what I’ll do.”

“If they are otherwise fit healthy people with no asthma or other particular indications to have the flu injection, I wouldn’t – I probably wouldn’t advise having it”.

“It’s not high on my priority. I think around March, when the flu vaccines come out, you tend to be much more likely to bring it up with patients, or they will bring it up with you.”

All general practitioners said that the majority of their pregnant patients were not aware that the influenza vaccine was recommended, and that most were initially reluctant to receive the vaccine due to safety concerns. They reported that only on rare occasions would a pregnant patient request the vaccine because they had heard about it elsewhere. The general practitioners who recommended the vaccine described their approach to the topic of influenza vaccination with their patients as involving an explanation of the risks of infection, benefits of vaccination, and reassurance that the vaccine is safe.

“They’re [pregnant women] always worried about side effects from it in terms of relating to the pregnancy but with a bit of explanation their fears can be allayed usually”.

“I think it’s more educating the patient because they’re not comfortable to have injection or anything during pregnancy but we have to offer it. I think I just have to convince them that given the pros and cons during pregnancy I think I have no problem. Initially I have some difficulty of convincing the pregnant lady to have the injection”.

Many of the general practitioners who recommend the vaccine reported that while they would advise the patient to be vaccinated according to the guidelines, they would ultimately leave the decision regarding vaccination to their patient. They saw influenza vaccination during pregnancy as a personal choice, and were not willing to strongly recommend it. These were predominantly the general practitioners who were not confident about the safety of the vaccine, and those who feared adverse outcomes or being blamed if there was an adverse outcome. One stated that they felt they could not be held liable for not recommending the vaccination.

“We do not push it. We do not insist. We just advise them. If they accept that's fine”.

“It’s a personal choice thing, so I don’t impose too much education on them at that stage because I’m not sure myself. I’m not fully convinced that it’s totally safe to them, no.”

“I'd even probably be relieved if they gave an indication that they weren't keen or felt a little bit uneasy, I would probably encourage that because then that's easier. (Then) there's no risk of anything going wrong with the vaccination. Kind of a ‘first do no harm’ kind of thing”.

The general practitioners who do recommend the vaccine to pregnant women reported varied rates of acceptance and uptake among their pregnant patients. Most general practitioners reported that “most” or “some” of their patients would agree to receive the vaccine once recommended, while a few reported that they had not yet convinced one pregnant patient to accept it. The general practitioners with more success identified a strong patient-doctor relationship and their patients having trust in them as important factors in patients accepting the vaccine, and some identified that as a general practitioner, they have significant power in convincing patients to accept the vaccine.

“I have a good relationship with the patients and if I recommend it they probably would take it on board”.

“I've got very convincing powers. They trust. I guess having a family doctor, they do trust.”

“They will take it positively if I am positive about it. If I say it is good they will see it as good. This is for the most part anyway”.

“It depends on how long you have the relationship as a doctor because I’m just new here so sometimes there’s some difficulty of getting them to trust me because they don’t know me”.

## Discussion

Many of the general practitioners interviewed in this study demonstrated limited knowledge about the risks of influenza infection and vaccination during pregnancy, or expressed limited confidence in the safety of the vaccine. These general practitioners may therefore either not be recommending the vaccine, or recommending it with varying levels of clarity and conviction. This may impact upon the fact that only a few general practitioners were able to report high acceptance of the vaccine among their pregnant patients. Our research findings concur with the results of previous studies which report that other antenatal care providers have varying levels of knowledge about influenza vaccination during pregnancy [22,28-30,36]. This is significant, as increased levels of provider knowledge are associated with higher rates of influenza vaccination in pregnant patients [28].

This is the first study to ascertain the attitudes, perceptions, and practices of Australian general practitioners towards influenza vaccination during pregnancy, and the qualitative methods used enabled us to capture rich information about their perspectives which could not have been garnered from a written survey. Semi-structured interviews were identified as the most appropriate data collection method to minimise participant burden and maximise the participation rate, as the interview could be conducted in the general practitioner’s workplace. It was anticipated that the participation rate for focus group discussions would be low. The limitation of this study is that only 17 of the 44 general practitioners invited to participate in the study agreed to participate. A greater number of interviews may have provided greater diversity of practice types. The responses of the general practitioners may have been affected by the fact that the interviewer is a NSW public health employee, however the findings did not indicate that they were reluctant to report practices and attitudes that were contrary to current guidelines.

In our study, general practitioners appear to base their overall risk assessment on their perceptions of the risk of influenza infection during pregnancy, the benefits and risks of the influenza vaccine, as well as their personal experiences of influenza. General practitioners appear to perceive the risks associated with maternal influenza infection to be lower than the evidence suggests, while conversely, their perception of the risks associated with the vaccine seem to be higher. Based on their risk assessments, general practitioners appear to be demonstrating two decision-making biases which influence whether and how they recommend the vaccine. Firstly, omission bias (choosing to do nothing with some probability of harm over doing something with a lower probability of equivalent harm) is demonstrated with general practitioners choosing to not recommend influenza vaccination, even though the probability of harm from influenza during pregnancy is higher than the probability of harm from the vaccine [15,37]. Omission bias is greater when practitioners anticipate that they will feel regret should an adverse outcome occur. However practitioners may anticipate a greater sense of regret if they perceive that the outcome could be the result of their own action rather than lack of action [38,39]. The general practitioners interviewed in our study clearly indicated anticipating regret (and fearing liability) should an adverse outcome occur due to the vaccine. Omission bias has been observed in parents deciding whether or not to vaccinate their child against pertussis, [40] and in practitioners deciding whether or not to recommend hormone replacement therapy for women [41]. Secondly, ambiguity bias (avoiding an option when information about the consequences is perceived to be missing [36]) is also observed here, with some general practitioners perceiving that more time is required to demonstrate that the use of the vaccine during pregnancy is safe. Both these decision-making biases may be limiting the extent to which the general practitioners provide influenza vaccination during pregnancy, and strategies aimed at improving the frequency of influenza vaccination to pregnant women by general practitioners should address these biases.

While the majority of the general practitioners reported intermittently recommending the vaccine, they reported varied levels of acceptance of the vaccine by their pregnant patients, which may be related to the quality, style, and consistency of the recommendation they give. This may in turn be related to our finding that these practitioners often lack confidence in their knowledge of the evidence, or in the safety of the vaccine for pregnant women. Many of the general practitioners reported that they are adopting the principles of patient informed choice – they are reluctant to provide a strong opinion or recommendation to their patients, often due to their own lack of confidence in the safety of the vaccine and their fear the consequences of liability should something go wrong. Those interviewed stated that they believe it is the patient’s decision whether they receive the vaccine or not, and pregnant women are therefore expected to decide for themselves. However, an informed decision requires relevant good quality information, [42] and it appears that pregnant women may not receive clear information from their general practitioner on the risks of influenza, and the benefits and risks of the vaccine. A qualitative survey of post-partum women in Switzerland indicated that they perceived that the information they received about vaccination from a provider, when given, lacked unequivocal advice and a sure recommendation [43]. Significant challenges have been reported regarding patient informed decision making - even when good information is provided patients face difficulties, especially when making complex decisions or when they are required to weigh benefits and risks [44]. In relation to influenza vaccination during pregnancy, women have indicated that they feel it is the physician who should explain the choice of whether to be vaccinated or not, and if the message was clear and unequivocal then they would follow the recommendation [42]. Even women who have safety concerns about the vaccine still indicate that they would accept it if the provider recommended it [23].

These interviews were undertaken approximately six months after the implementation of a number of strategies aimed to increase awareness of maternal influenza vaccination during pregnancy among general practitioners, including a letter and a brochure, however only a small number of interviewees recalled the information contained in these documents. A survey of post-partum women in the same region conducted concurrently with this study found the rate of vaccine uptake among pregnant women to be 25% during the 2012 influenza season, and that only 30% of women could recall receiving a recommendation for the vaccine from an antenatal health care provider during their pregnancy [45]. This suggests that the strategies undertaken were not sufficient to increase rates of provider recommendation and vaccine uptake to acceptable levels. Different strategies may be required that more clearly address the risk perceptions of general practitioners identified in this study, and as many interviewees raised the issue of requiring more time to increase their confidence, it may be expected that changes in practice regarding this issue may not occur quickly.

We have not located any research that has investigated how to improve rates of vaccination recommendation in general practitioners across a region; however a number of studies conducted at single antenatal clinic sites have demonstrated that it is possible to increase provider knowledge about influenza vaccination during pregnancy, rates of provider recommendation or patient acceptance of the vaccine through strategies targeting providers, pregnant women, or both. Strategies targeting women include displaying posters in the clinics and providing patients with information brochures, and strategies targeting providers include provider education programs, reminder stamps in patient’s files, e-mail reminder to providers, and making the vaccine available in the clinic [21,46,47]. In an obstetric hospital in Melbourne the implementation of a combination of these strategies saw influenza vaccination coverage increase from 30 to 40%, and rates of provider recommendation increasing from 37% to 62.5% [21]. The inclusion of an electronic best practice alert about influenza vaccination in each patient’s electronic medical record in an antenatal clinic in Wisconsin saw vaccination coverage improve from 42% to 61% and provider recommendation rates improve from 49 to 89% [48]. This strategy could translate well to the general practice context in Australia, where electronic decision support for cardiovascular disease management in general practice shows promising potential to increase provider adherence to best practice guidelines [49]. A number of general practitioners in this study reported that influenza vaccination during pregnancy is not always their key priority and they often forget to mention it, and some suggested an electronic reminder could assist with this. Others also recommended that if women were more aware and knowledgeable about the vaccination it would make their job easier to convince them, and suggested that a public awareness campaign could assist with this.

## Conclusion

General practitioners have varied knowledge, attitudes and beliefs about influenza vaccination during pregnancy and this is likely to influence both the number of recommendations given to pregnant women, and the conviction with which these recommendations are made. This study indicated that general practitioners have a low perception of risk of influenza during pregnancy, and have considerable concerns about vaccine safety and potential liability, and additional research could further investigate these issues. Providing clear information and communication to general practitioners on the current recommendations regarding influenza vaccination during pregnancy, and the evidence upon which they are made, may increase provider knowledge and confidence in recommending the vaccine, which is likely to improve influenza vaccine coverage among Australian pregnant women.

## Competing interests

The authors declare that they have no competing interests.

## Authors’ contribution

LM, SC, and KH participated in the study design, co-ordination, and implementation. LM conducted the interviews. LM, KH, KW, and AD conducted the analysis. LM drafted the manuscript. ST and GL participated in project design and oversaw implementation. All authors read and approved the final manuscript.

## Pre-publication history

The pre-publication history for this paper can be accessed here:

http://www.biomedcentral.com/1471-2296/15/102/prepub

## References

[B1] Rothberg HB Haessler SD Brown RB Complications of viral influenza Am J Med 2008 121 4 258 264 10.1016/j.amjmed.2007.10.040 18374680 PMC7172971

[B2] Neuzil KM Reed GW Mitchel EF Simonsen L Griffin MR Impact of influenza on acute cardiopulmonary hospitalizations in pregnant women Am J Epidemiol 1998 148 11 1094 1102 10.1093/oxfordjournals.aje.a009587 9850132

[B3] Jamieson DJ Honein MA Rasmussen SA Williams JL Swerdlow DL Biggerstaff MS Lindstrom S Louie KJ Christ CM Bohm SR Fonseca VP Ritger KA Kuhles DJ Eggers P Bruce H Davidson HA Lutterloh E Harris ML Burke C Cocoros N Finelli L MacFarlane KF Shu B Olsen SJ Novel Influenza A (H1N1) Pregnancy Working Group H1N1 2009 influenza virus infection during pregnancy in the USA Lancet 2009 374 9688 451 458 10.1016/S0140-6736(09)61304-0 19643469

[B4] Cox S Posner SF McPheeters M Jamieson DJ Kourtis AP Meikle S Hospitalisation with respiratory illness among pregnant women during Influenza season Obstet Gynecol 2006 107 1315 1322 10.1097/01.AOG.0000218702.92005.bb 16738158

[B5] Webb SA Pettila V Seppelt I Bellomo R Bailey M Cooper DJ Cretikos M Davies AR Finfer S Harrigan PW Hart GK Howe B Iredell JR McArthur C Mitchell I Morrison S Nichol AD Paterson DL Peake S Richards B Stephens D Turner A Yung M ANZIC Influenza Investigators Critical Care Services and 2009 H1N1 Influenza in Australia and New Zealand New Engl J Med 2009 361 1925 1934 19815860 10.1056/NEJMoa0908481

[B6] Hewagama S Walker SP Stuart RL Gordon C Johnson PD Friedman ND O’Reilly M Cheng AC Giles ML 2009 H1N1 influenza a and pregnancy outcomes in Victoria Australia Clin Infect Dis 2010 50 5 686 689 10.1086/650460 20100064

[B7] Webb SA Pettila V Seppelt I Bellomo R Bailey M Cooper DJ Cretikos M Davies AR Finfer S Harrigan PW Hart GK Howe B Iredell JR McArthur C Mitchell I Morrison S Nichol AD Paterson DL Peake S Richards B Stephens D Turner A Yung M ANZIC Influenza Investigators Critical illness due to 2009 A/H1N1 influenza in pregnant and postpartum women: population based cohort study BMJ 2010 340 7749 751

[B8] Rasmussen SA Jamieson DJ Bresee JS Pandemic influenza and pregnant women Emerg Infect Dis 2008 14 1 95 10.3201/eid1401.070667 18258087 PMC2600164

[B9] Zaman K Eliza R Shams E Arifeen S Rahman R Raqib R Wilson E Omer S Shahid N Breiman R Effectiveness of maternal influenza immunization in mothers and infants New Engl J Med 2008 359 1555 1564 10.1056/NEJMoa0708630 18799552

[B10] Benowitz I Esposito DB Gracey KD Shapiro ED Vázquez M Influenza vaccine given to pregnant women reduces hospitalization due to Influenza in their infants Clin Infect Dis 2010 51 12 1355 1361 10.1086/657309 21058908 PMC3106242

[B11] Eick AA Uyeki T Klimov A Hall H Reid R Santosham M O’Brien K Maternal influenza vaccination and effect on influenza virus infection in young infants Arch Pediat Adol Med 2011 165 2 104 111 10.1001/archpediatrics.2010.192 20921345

[B12] Tamma PD Ault KA Del Rio C Steinhoff MC Halsey NA Omer SB Safety in Influenza vaccination during pregnancy Am J Obstet and Gynecol 2009 201 6 547 552 10.1016/j.ajog.2009.09.034 19850275

[B13] Moro PL Border K Zheteyeva Y Revzina N Tepper N Kissin D Barash F Arana J Brantley M Ding H Singleton J Walton K Haber P Lewis P Yue X DeStefano F Vellozzi C Adverse events in pregnant women following administration of trivalent inactivated Influenza vaccine and live attenuated Influenza vaccine in the vaccine adverse event reporting system, 1990–2009 Am J Obstet and Gynecol 2011 204 2 146.e1 146.e7 20965490 10.1016/j.ajog.2010.08.050

[B14] Moro PL Broder K Zheteyeva Y Revzina N Tepper N Kissin D Barash F Arana J Brantley M Ding H Singleton J Walton K Haber P Lewis P Yue X DeStefano F Vellozzi C Adverse events following administration to pregnant women of influenza A (H1N1) 2009 monovalent vaccine reported to the vaccine adverse event reporting system Am J Obstet Gynecol 2011 205 473 e1 e9 21861964 10.1016/j.ajog.2011.06.047 PMC6602056

[B15] Haberg S Trogstad L Gubbes N Wilcox A Gjessing H Samuelsen S Skrondal A Cappelen I Engeland A Aavitsland P Madsen S Buajordet I Furu K Nafstad P Vollset SE Feiring B Nøkleby H Magnus P Stoltenberg C Risk of fetal death after pandemic influenza virus infection or vaccination New Engl J Med 2013 368 333 340 doi:10.1056/NEJMoa1207210 10.1056/NEJMoa1207210 23323868 PMC3602844

[B16] National Health and Medical Research Council The Australian immunisation handbook 2013 10 Australia: NHMRC

[B17] NSW Health Influenza information for antenatal care providers 2013 http://www.health.nsw.gov.au/Infectious/Influenza/Pages/hp_pregnancy_advice.aspx

[B18] Royal Australian and New Zealand College of Obstetrics and Gynaecology Influenza vaccination for pregnant women 2013 http://www.ranzcog.edu.au/component/docman/doc_download/978-influenza-vaccination-for-pregnant-women-c-obs-45.html?Itemid=946; 2013. Accessed 28/05/2014

[B19] Mak DB Daly AM Armstrong PK Effler PV Pandemic (H1N1) 2009 influenza vaccination coverage in Western Australia Med J Australia 2010 193 7 401 404 20919971 10.5694/j.1326-5377.2010.tb03969.x

[B20] White S Petersen R Quinlivan J Pandemic (H1N1) 2009 influenza vaccine uptake in pregnant women entering the 2010 influenza season in Western Australia Med J Australia 2010 193 405 407 20919972 10.5694/j.1326-5377.2010.tb03970.x

[B21] McCarthy EA Pollock WE Nolan T Hay S McDonald S Improving influenza vaccination coverage in pregnancy in Melbourne 2010–2011 Aust N Z J Obstet Gynaecol 2012 52 4 334 341 doi:10.1111/j.1479-828X.2012.01428.x 10.1111/j.1479-828X.2012.01428.x 22486173

[B22] Lu AB Halim AA Dendle C Kotsanas D Giles ML Wallace EM Buttery JP Stuart RL Influenza vaccination uptake amongst pregnant women and maternal care providers is suboptimal Vaccine 2012 30 4055 4059 10.1016/j.vaccine.2012.04.012 22521842

[B23] Wiley K Massey PD Cooper Robbins SC Wood N Ho J Quinn HE Leask J Uptake of influenza vaccine by pregnant women: a cross-sectional survey with implications for policy and practice Med J Aust 2013 198 7 373 375 10.5694/mja12.11849 23581957

[B24] Blanchard-Rohner G Meier S Ryser J Schaller D Combescure C Yudin MH Burton-Jeangros C De Tejada BM Siegrist CA Acceptability of maternal immunization against influenza: the critical role of obstetricians J Matern Fetal Neonatal Med 2012 25 9 1800 1809 doi:10.3109/14767058.2012.663835. Epub 2012 Mar 16 10.3109/14767058.2012.663835 22339083

[B25] Dlugacz Y Fleischer A Carney MT Copperman N Ahmed I Ross Z Buchman T Fried A Cabello C De Geronimo M Sweetapple C Besthoff CM Silverman RA 2009 H1N1 vaccination by pregnant women during the 2009–10 H1N1 influenza pandemic Am J Obstet Gynecol 2012 206 339 e1 e8 22306303 10.1016/j.ajog.2011.12.027

[B26] Lau J Caia Y Tsuia H Choia K Prevalence of influenza vaccination and associated factors among pregnant women in Hong Kong Vaccine 2010 28 5389 5397 10.1016/j.vaccine.2010.05.071 20542072

[B27] Ding H Santibanez T Jamieson D Weinbaum C Euler G Grohskopf L Lu P Singleton J Influenza vaccination coverage among pregnant women–National 2009 H1N1 flu survey (NHFS) Am J Obstet Gynecol 2011 204 S96 S106 21640233 10.1016/j.ajog.2011.03.003

[B28] Eppes C Wu A Cameron K Garcia P Grobman W Does obstetrician knowledge regarding influenza increase HINI vaccine acceptance among their pregnant patients? Vaccine 2012 2012 30 5782 5784 22743135 10.1016/j.vaccine.2012.06.032

[B29] Tong A Biringer A Ofner-Agostini M Upshur R McGeer A A cross-sectional study of maternity care provider’s and women’s knowledge: attitudes, and behaviors towards influenza vaccination during pregnancy J Obstet Gynaecol Can 2008 30 5 404 410 18505664 10.1016/s1701-2163(16)32825-0

[B30] Kissin D Power M Kahn E Williams J Jamieson D MacFarlane K Schulkin J Zhang Y Callaghan W Attitudes and practices of obstetrician–gynecologists regarding influenza vaccination in pregnancy Obstet Gynecol 2011 118 5 1074 1080 10.1097/AOG.0b013e3182329681 22015875 PMC4608446

[B31] NSW Ministry of Health Influenza vaccination during pregnancy. Protect you and your baby from influenza (flu) 2013 Brochure Available at http://www.health.nsw.gov.au/Infectious/Influenza/Documents/web-eng-flyer.pdf. Accessed 28/05/2014

[B32] Neergard MA Olesen F Andersen RS Sondergaard J Qualitative description – the poor cousin of health research? BMC Med Res Methodol 2009 9 52 doi:10.1186/1471-2288-9-52 10.1186/1471-2288-9-52 PMC2717117 19607668

[B33] Trost JE Statistically non-representative stratified sampling: a sampling technique for qualitative studies Qualitative Sociology 1986 9 1 54 57 10.1007/BF00988249

[B34] Lincoln YS Guba EG Naturalistic Inquiry 1985 Beverly Hills, CA: Sage 347 351

[B35] Department of Health and Aging Prescribing Medicines in Pregnancy Database 2012 http://www.tga.gov.au/hp/medicines-pregnancy.htm. Accessed 28/05/2014

[B36] Broughton D Beigi R Switzer G Raker C Anderson B Obstetric health care workers’ attitudes and beliefs regarding influenza vaccination in pregnancy Obstet Gynecol 2009 114 5 981 987 10.1097/AOG.0b013e3181bd89c2 20168097

[B37] Ritov I Baron J Reluctance to vaccinate: omission bias and ambiguity J Behav Decis Making 1990 3 263 277 10.1002/bdm.3960030404

[B38] Ritov I Outcome knowledge, regret and omission bias Organ Behav Hum Dec 1996 64 2 119 127

[B39] Bornstein B Emler C Rationality in medical decision making: a review of the literature on doctors’ decision making biases J Eval Clin Pract 2001 7 2 97 107 10.1046/j.1365-2753.2001.00284.x 11489035

[B40] Asch D Baron J Hershey JC Kunreauther HM Meszaros J Ritov I Spranca M Determinants of resistance to pertussis vaccination Med Decis Making 1994 14 118 123 10.1177/0272989X9401400204 8028464

[B41] Baron J Holzman G Schulkin J Attitudes of obstetricians and gynecologists toward hormone replacement therapy Med Decis Making 1998 18 406 doi:10.1177/0272989X9801800408 10.1177/0272989X9801800408 10372583

[B42] Marteau T Dormandy E Michie S A measure of informed choice Health Expect 2001 4 99 108 10.1046/j.1369-6513.2001.00140.x 11359540 PMC5060053

[B43] Schindler M Blanchard-Rohner G Meier S Martinezde Tejada B Siegrist C-A Burton-Jeangros vaccination against seasonal flu in Switzerland: the indecision of pregnant women encouraged by healthcare professionals Rev Epidemiol Sante 2012 60 447 453 10.1016/j.respe.2012.03.008 23141298

[B44] Woolf S Chan E Harris R Sheridan S Braddock C Kaplan R Krist A O’Connor A Tunis S Promoting informed choice: transforming health care to dispense knowledge for decision making Ann Intern Med 2005 143 293 300 10.7326/0003-4819-143-4-200508160-00010 16103473

[B45] Maher L Hope K Torvaldsen S Lawrence G Dawson A Wiley K Thomson D Hayen A Conaty S Influenza vaccination during pregnancy: coverage rates and influencing factors in two urban districts in Sydney Vaccine 2013 31 5557 5564 10.1016/j.vaccine.2013.08.081 24076176

[B46] Panda B Stiller R Panda A Influenza vaccination during pregnancy and factors for lacking compliance with current CDC guidelines J Matern-Fetal Neo M 2011 24 3 402 406 10.3109/14767058.2010.497882 20593974

[B47] Yudin H Salripour M Sgro M Impact of patient education on knowledge of influenza and vaccine recommendations among pregnant women J Obstet Gynaecol Can 2009 32 3 232 237 20500967 10.1016/s1701-2163(16)34449-8

[B48] Klatt T Hopp E Effect of a best-practice alert on the rate of influenza vaccination of pregnant women Obstet Gynecol 2012 119 2 Pt 1 301 305 doi:10.1097/AOG.0b013e318242032a 22270281 10.1097/AOG.0b013e318242032a

[B49] Peiris D Joshi R Webster R Groenestein P Usherwood T Heeley E Turnbull F Lipman A Patel A An electronic clinical decision support tool to assist primary care providers in cardiovascular disease risk management: development and mixed methods evaluation J Med Internet Res 2009 11 4 e51 10.2196/jmir.1258 20018588 PMC2802562

